# Mitigating salinity and cadmium stress in rice (*Oryza sativa* L.) using PGPR and salicylic acid: rhizosphere, health risk, and physiological insights

**DOI:** 10.1080/15592324.2025.2553803

**Published:** 2025-09-06

**Authors:** Arwa Abdulkreem Al-Huqail, Muna Abdul-Rahman Al-Malki, Dalia Mohammad Melebari, Hanan El Sayed Osman, Dikhnah Alshehri, Suliman Mohammed Suliman Alghanem, Amany H.A. Abeed, Hesam Mousavi

**Affiliations:** a Department of Biology, College of Science, Princess Nourah bint Abdulrahman University, Riyadh, Saudi Arabia; b Biology Department, College of Science, Umm Al-Qura University, Makkah, Saudi Arabia; c Botany and Microbiology Department, Faculty of Science, Al-Azhar University, Cairo, Egypt; d Department of Biology, Faculty of Science, University of Tabuk, Tabuk, Saudi Arabia; e Department of Biology, College of Science, Qassim University, Burydah, Saudi Arabia; f Botany and Microbiology Department, Faculty of Science, Assiut University, Assiut, Egypt; g Faculty of Applied Ecology, Agricultural Science and Biotechnology, University of Inland Norway, Elverum, Norway

**Keywords:** Salt stress, metal accumulation, acids, growth bacteria, gene expression, proteomics

## Abstract

Soil contamination with salinity and heavy metals such as cadmium (Cd) is becoming a serious global problem due to the rapid development of the social economy. Although plant growth-promoting rhizobacteria PGPR and organic agents such as salicylic acid (SA) are considered major protectants to alleviate abiotic stresses, the study of these bacteria and organic acids to ameliorate the toxic effects of salinity and Cd remains limited. Therefore, the present study was conducted to investigate the individual and combined effects of PGPR and SA on enhancing the phytoremediation of salinity (100 mM NaCl) and Cd (50 µM CdCl₂) using rice (*Oryza sativa* L.) plants. The research results indicated that elevated levels of salinity and Cd stress in soil significantly (*P* < 0.05) decreased plant growth and biomass, photosynthetic pigments, and gas exchange attributes. However, salinity and Cd stress also induced oxidative stress in the plants by increasing malondialdehyde (MDA) and hydrogen peroxide (H_2_O_2_) by 44% and 38%, respectively, which also induced increased compounds of various enzymatic and nonenzymatic antioxidants, and also the gene expression and sugar content. Furthermore, a significant (*P* < 0.05) increase in cadmium accumulation, potential health risk indices, proline metabolism, the AsA–GSH cycle, and the pigmentation of cellular components was observed. Although the application of PGPR and SA showed a significant (*P* < 0.05) increase in plant growth and biomass, gas exchange characteristics, microbial diversity, functional gene abundance in the rhizosphere, enzymatic and nonenzymatic compounds, and their gene expression, and also decreased oxidative stress. In addition, the application of PGPR and SA enhanced cellular fractionation and decreased metal accumulation by 37% in shoots, proline metabolism, and the AsA–GSH cycle in *O. sativa* plants. These results provide new insights for sustainable agricultural practices and hold immense promise in addressing the pressing challenges of salinity and heavy metal contamination in agricultural soils.

## Graphical abstract

**Figure:**
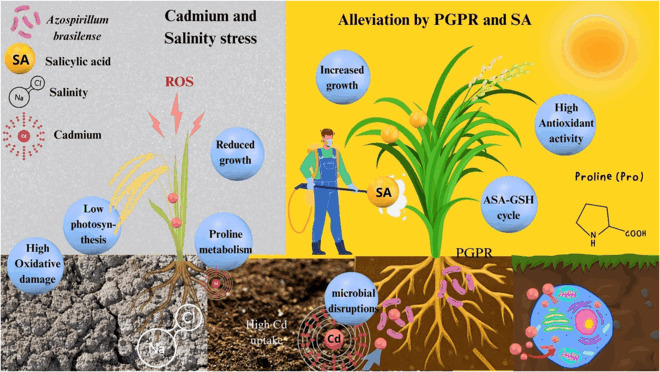

## Introduction

Environmental variations due to abiotic stresses, such as drought, heat, cold, salinity, and metal toxicity, adversely affect and limit agricultural productivity.^1^^,^^2^ Salinization of arable soil is one of the key environmental bottlenecks that affects agricultural productivity worldwide.^3^ The salinity issue affects as much as 20% of irrigated lands worldwide, hindering plant growth due to osmotic stress, ion toxicity, and nutritional imbalance disorders.^4^ It significantly impacts photosynthesis, enzyme activity, and hormone regulation, which in turn negatively influences crop yields.^5^ Multiple studies have shown that salinity leads to excessive accumulation of reactive oxygen species (ROS), causing oxidative damage throughout all levels of cellular structures.^6^ For instance, Ahmad et al.^7^ reported that salt stress has a significant disruptive effect on the antioxidant defense system in rice. Nonetheless, to overcome this issue, several studies have emphasized the need for breeding and biotechnological approaches to improve salt tolerance in crops.^8-11^ However, salinity remains a persistent issue despite various efforts, given the complex physiological and molecular responses it triggers within the plants.

In addition to salinity, another critical and destructive issue affecting agriculture is heavy metal pollution.^12-14^ Among all heavy metals, cadmium (Cd) attained more research interest because of its unique properties, such as higher solubility and mobility in soil‒plant systems,^15^ together with the fact that it is toxic to plants even at very low concentrations.^16^ Photosynthesis, respiration, cell division, water relations, opening and closing of stomata, nitrogen metabolism, and mineral nutrition are the main metabolic processes within the plants that are negatively affected by Cd stress.^17^ Although Cd is toxic for plant growth, it is easily taken up by roots and then transported to shoots, where it can cause retorted growth, stunted root development, reduced branching, alteration in photosynthesis and respiration, diminished nutrient uptake, blocked electron transport chain, as well as changed the membrane permeability.^18^ Moreover, higher Cd retention in plant cells/tissues triggers the production of reactive oxygen species (ROS), hydroxyl groups (OH), and superoxide radicals (O^.−^), which either directly or indirectly affect the in planta metabolic pathways.^19^ Overproduction of ROS is toxic, and plants need to scavenge them immediately through antioxidative defense system.^20^

Recent advancements in plant biotechnology have underscored the synergistic potential of organic acids and beneficial plant growth-promoting rhizobacteria (PGPR) in mitigating salinity and heavy metal toxicity.^12^^,^^21-23^ The use of organic acids has grown rapidly due to their broad application across agriculture and industrial domains.^22^^,^^24^ Among various options, the use of salicylic acid (SA) is becoming increasingly widespread due to its numerous advantages over chemical fertilization.^25^ SA is recognized for its role in enhancing nutrient uptake, mitigating oxidative damage, and modulating physiological and biochemical processes under salinity and metal stress conditions, thereby promoting sustainable plant growth.^26^ Previous studies have shown that using SA can reduce the toxic effects of metals in *T. aestivum* grown in Cd-contaminated soil.^27^ Similarly, plant growth-promoting rhizobacteria (PGPR) help in improving plant growth, salinity, and metal resistance^28^^,^^29^ by modifying the concentration of growth regulators and phytohormones that facilitate the plant's ability to tolerate environmental stresses.^30^ PGPR enhances Cd and salinity bioremediation by improving heavy metal bioavailability, uptake, and conversion into less toxic forms through methylation, oxidation, demethylation, and reduction.^28^^,^^31^*Azospirillum brasilense* is a well-known PGPR with significant capabilities in promoting plant growth. ^32^ This bacterium has been found to enhance plant resilience by modulating physiological and biochemical processes, particularly under stressed environments.^27^ The application of this bacterium under stressed conditions has shown promising results in boosting plant growth, increasing nutrient absorption, and reducing oxidative stress.^33^ Moreover, recent studies have confirmed the effectiveness of *A. brasilense* in alleviating the adverse impacts of environmental factors, including salinity, drought, and toxicity from heavy metals in wheat.^34^

In many agricultural regions, salinity and cadmium contamination frequently co-occur, exerting compounded stress on crops. Rice (*Oryza sativa* L.) grown in saline and Cd-contaminated soils challenges food production and quality. Therefore, there is an increasing demand to simultaneously minimize Cd accumulation and alleviate salinity stress in *O. sativa* production and food safety. However, the application of crop residues, manure, compost, fertilizers, micronutrients, and biochar are among the organic amendments commonly used under conditions of Cd toxicity and soil salinity.^23^ Although the individual roles of PGPR and SA have been studied under salinity or Cd stress separately, their combined impact on rice under both salinity and Cd stress remains insufficiently explored, particularly in relation to molecular, proteomic, and microbial dynamics. Moreover, most previous studies have focused on either salinity or cadmium stress in isolation, often neglecting the complex and synergistic effects that arise under their combined occurrence, as well as the integration of physiological, molecular, and rhizospheric responses. Therefore, the present study was conducted to study (1) the effects of PGPR and organic acids on plant growth, biomass, and gaseous exchange parameters of *O. sativa* under salinity and Cd stress, (2) oxidative stress and the responses of different antioxidative enzymes (enzymatic and nonenzymatic), as well as the response of specific gene expression, (3) proline metabolism, AsA–GSH cycle and cellular fractionation, and Cd accumulation in different organs of *O. sativa* under Cd stress, and also (4) the influence of these treatments on rhizosphere microbiome composition and diversity, as well as the evaluation of potential human health risk through health risk index analysis. The results from the present study provided new insights that the use of PGPR and organic acids in salinity and heavy metal studies may be beneficial and can improve plant yield in contaminated soils.

## Materials and methods

**Figure 1.**
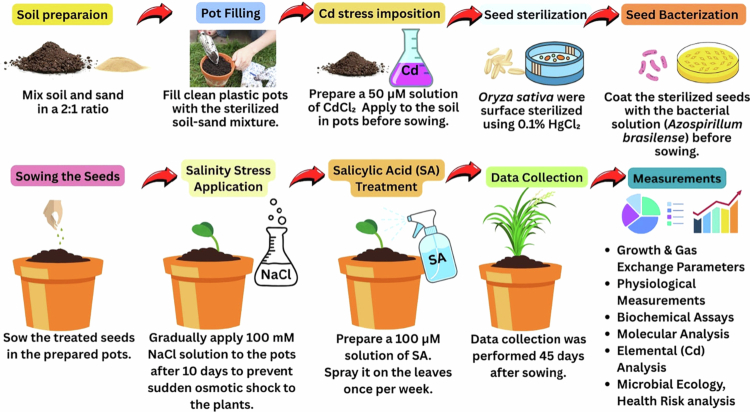
Methodology for conducting experiments to evaluate how salicylic acid (SA) and PGPR (*Azospirillum brasilense*) interact with *Oryza sativa* under individual and combined salinity (100 mM NaCl) and cadmium (50 µM CdCl₂) stress. The workflow includes rhizospheric soil collection and preparation, followed by amendment with specific stressors and treatments. Rice seeds were surface sterilized and sown in sterilized soil–sand mixture (2:1). Treatments were applied after 10 d of germination, and the plants were maintained under controlled greenhouse conditions. After 45 d, the plants were harvested and analyzed for growth, physiological traits, gas exchange parameters, oxidative stress markers, antioxidants (enzymatic and nonenzymatic), secondary metabolites, Cd accumulation, rhizosphere microbial diversity, and molecular (qRT‒PCR) and proteomic (SDS‒PAGE, mass spectrometry) responses.

Healthy seeds of rice (*O. sativa* L.) were surface sterilized with 10% (v/v) commercial bleach for 10–20 min and then washed with distilled water. A pot experiment was conducted in the Botanical Garden under the glasshouse. Pots were placed in a glasshouse environment where they received natural sunlight, day/night humidity (60/70%), and day/night temperature (24/12°C), respectively. Uncontaminated soil, obtained from the research field, was air-dried and passed through a 2-mm sieve. The initial physicochemical properties of natural soil were as follows: pH − 6.9, EC − 0.9 dS cm^−1^, organic matter − 17 g kg^−1^, EK − 21 mg kg^−1^, TP − 0.17 g kg^−^^1^, and TN − 16 g kg^−^^1^. After contamination of soil with salinity stress by irrigating with a 100 mM NaCl and Cd stress using cadmium chloride (CdCl_2_) at a level of 50 µM in the soil, the pots (30-cm-tall * 40-cm-wide) were filled with 10 kg of amended soil and subjected to four different cycles of water, equilibrated for two months, and then air dried. SA was applied to the *O. sativa* plants after the seedling stage, 14 d after seed sowing. Organic acid was added as an aqueous solution at a concentration of SA (100 µM) and applied as a foliar spray to the *O. sativa's* seedlings. The foliar spray with SA was provided twice a week, and the control was provided by de-ionized water. Regarding the PGPR, i.e., *A. brasilense*, we have followed the method presented by Cui et al.^35^ The bacterial isolates were biochemically characterized based on the protocols outlined in “Bergey's Manual of Determinative Bacteriology” by Holt et al.^36^ The pots used in this study were rotated regularly to avoid environmental effects on the plants. A complete randomized design (CRD) with four replications was used. The total duration of the experimental treatments was 3 months under controlled conditions. A detailed schematic presentation of the entire methodology is provided in Figure 1.

### Procurement and characteristics of bacterial strain

Bacterial isolation was carried out through the pore plate method.^37^ Briefly, 100 µL of 106 times serially diluted soil solution (prepared in physiological saline) was spread on Luria–Bertani (LB) media plates and incubated for 24 h at 30°C. Purified bacterial strains were evaluated for their tolerance to salinity and Cd stress in terms of maximum inhibitory concentration (MIC) by gradually increasing salinity and Cd concentration in LB plates until the bacterial isolates failed to grow. This concentration was considered the MIC of the respective isolate. Among the isolated salinity- and Cd-resistant bacteria *A. brasilense* was found highly salt resistant (MIC = 60 g L^−1^ Cd) and efficient among the other isolates in studied plant growth-promoting activities under stress conditions. Therefore, the bacterial strain *A. brasilense* was further characterized and evaluated in pot experiment followed by Cui et al.,^37^ to test *A. brasilense* under salinity and Cd stress. The raw sequencing data of the microbial community have been deposited in the NCBI Sequence Read Archive (SRA) under the accession number PRJNA123456.

### Harvesting and data collection

After three months, the remaining seedlings were uprooted and washed gently with the help of distilled water to eliminate the aerial dust and deposition. Functional leaf in each treatment was picked at a rapid growth stage during 09:00–10:30 a.m. The sampled leaves were washed with distilled water, immediately placed in liquid nitrogen, and stored in a freezer at −80°C for further analysis. All the harvested plants were divided into two parts, i.e., roots and shoots to study different physio-biochemical traits. Leaves from each treatment group were collected for chlorophyll, oxidative stress, and antioxidants analysis. Root and shoot lengths were measured straightway after the harvesting by using measuring scale and a digital weighing balance to measure fresh biomass. Roots were uprooted and immersed in 20 mM Na_2_EDTA for 15–20 min to remove salinity and Cd adhered to the root surfaces. Then, roots were washed thrice with distilled water and finally once with de-ionized water and dried for further analysis. The different parts of the plant (roots and shoots) were oven-dehydrated at 65°C for 72 h for Cd determination, and the total plant dry weight was also measured.

### Measurement of chlorophyll and carotenoid content and gas exchange traits

For chlorophyll content analysis, 0.1 g of fresh leaf sample was extracted with 8 mL of 95% acetone for 24h at 4°C in the dark. The absorbance of the resulting solution was measured by a spectrophotometer (UV-2550; Shimadzu, Kyoto, Japan) at 646.6, 663.6, and 450 nm. The chlorophyll content was then calculated using the standard method described by Arnon and Whatley,^38^ ensuring that the final volume of each sample, after adding the supernatant to 85% acetone, was adjusted to 10 mL. On the same days, gaseous exchange was also measured. Net photosynthesis (Pn), leaf stomatal conductance (Gs), transpiration rate (Ts), and intercellular carbon dioxide concentration (Ci) were measured from three different plants in each treatment group. Measurements were taken from 9:00 a.m. to 11:00 a.m. to ensure consistent environmental conditions with a clear sky. The rates of leaf Pn, Gs, Ts, and Ci were measured with an LI-COR gas-exchange system (LI6400; LICOR Bio sciences, Lincoln, NE, USA) with a red blue LED light source on the leaf chamber. In the LI-COR cuvette, the CO_2_ concentration was set as 380 μmol mol^−^^1^, and the LED light intensity was set at 1000 mmol m^−2^ s^−1^, which is the average saturation intensity for photosynthesis in *O. sativa*.^39^

### Determination of antioxidants and proline metabolism

To evaluate enzyme activities, fresh leaves (0.5 g) were homogenized in liquid nitrogen and 5 mL of 50 mmol sodium phosphate buffer (pH 7.0), including 0.5 mmol EDTA and 0.15 mol NaCl. The homogenate was centrifuged at 12,000 × *g* for 10 min at 4°C, and the supernatant was used for the measurement of superoxidase dismutase (SOD) and peroxidase (POD) activities. SOD activity was assayed in 3 mL reaction mixture containing 50 mM sodium phosphate buffer (pH 7), 56 mM nitro blue tetrazolium, 1.17 mM riboflavin, 10 mM methionine, and 100  μL of enzyme extract. Finally, the sample was measured by using a spectrophotometer (xMark™ microplate absorbance spectrophotometer; Bio-Rad). Enzyme activity was measured using a method by Chen and Pan.^40^ Peroxidase activity in the leaves and roots was estimated using the method of Sakharov and Ardila^41^ using guaiacol as the substrate. A reaction mixture (3 mL) containing 0.05  mL of enzyme extract, 2.75  mL of 50  mM phosphate buffer (pH 7.0), 0.1  mL of 1% H_2_O_2_, and 0.1  mL of 4% guaiacol solution was prepared. An increase in the absorbance at 470 nm because of guaiacol oxidation was recorded for 2 min. One unit of enzyme activity was defined as the amount of the enzyme. Catalase (CAT) activity was analyzed according to Aebi.^42^ The assay mixture (3.0  mL) was comprised of 100 μL of enzyme extract, 100  μL of H_2_O_2_ (300  mM), and 2.8  mL of 50  mM phosphate buffer with 2 mM ETDA (pH 7.0). The CAT activity was measured from the decrease in absorbance at 240 nm as a result of H_2_O_2_ loss (*ε* = 39.4 mM^−^^1^ cm^−^^1^). Ascorbate peroxidase (APX) activity was measured according to Nakano and Asada.^43^ A mixture containing 100 μL of enzyme extract, 100 μL of ascorbate (7.5 mM), 100 μL of H_2_O_2_ (300 mM), and 2.7 mL of 25 mM potassium phosphate buffer with 2 mM EDTA (pH 7.0) was used for measuring APX activity. The oxidation pattern of ascorbate was estimated from the variations in wavelength at 290 nm (*ε* = 2.8 mM^−^^1^ cm^−^^1^).

A quantitative real-time PCR (RT‒qPCR) assay was applied to investigate the expression levels of 4 stress-related genes, including Fe-SOD, POD, CAT, and APX. Total RNA was extracted from leaf tissue samples using RNeasy Plant Mini kits (Qiagen, Manchester, UK). The RT-qPCR was performed using an Applied Biosystems 7500 Fast Real-Time PCR System with the following cycling parameters: initial denaturation at 95°C for 5 min, followed by 40 cycles of denaturation at 95°C for 15 s, annealing at 58°C−60°C (optimized for each primer pair) for 30 s, and extension at 72°C to 95°C for 30 s. A melting curve analysis was conducted from 65°C to confirm the specificity of the amplification. Contaminating DNA was then removed, and first-strand cDNAs were prepared using Reverse Transcription kits (Qiagen, Manchester, UK). RT‒qPCR analysis was conducted as reported in the protocol of QuantiTect SYBR Green PCR Kit (Qiagen, Manchester, UK). Reaction volume and PCR amplification conditions were adjusted as mentioned by El-Esawi et al.^44^

To measure proline concentrations, 0.5 g of shoot tissue was ground in sulfosalicylic acid and then centrifuged, and the supernatant was collected from each sample. The proline concentration in each sample was measured.^45^ Specifically, the supernatant from each sample was reacted with acid ninhydrin, and the resulting colorimetric reaction was measured to determine the proline concentration by “UV-1700 pharmaSpec spectrophotometer.” The ProDH “proline dehydrogenase,” P5CR “pyrroline-5-carboxylate reductase,” and P5C “pyrroline-5-carboxylate” were measured using kits provided by Jiangsu Meibiao Biological Technology Co., Ltd. Enzyme activities were accurately measured using these reagent kits, which include all the chemicals and related instructions by “UV-1700 pharmaSpec spectrophotometer.”

### Nonenzymatic compounds and sugar determination

Plant ethanol extracts were prepared for the determination of nonenzymatic antioxidants and some key osmolytes. For this purpose, 50 mg of dry plant material was homogenized with 10 mL of ethanol (80%) and filtered through Whatman No. 41 filter paper. The residue was re-extracted with ethanol, and the 2 extracts were pooled together to a final volume of 20 mL. The determination of flavonoids,^46^ phenolics,^47^ ascorbic acid,^48^ anthocyanin,^49^ and total sugars^50^ was performed from the extracts. Fresh leaf material (0.1 g) was mixed thoroughly in 5 mL of aqueous sulfosalicylic acid (3%). The mixture was centrifuged at 10,000 × *g* for 15 min, and an aliquot (1 mL) was poured into a test tube having 1 mL of acidic ninhydrin and 1 mL of glacial acetic acid. The reaction mixture was first heated at 100°C for 10 min and then cooled in an ice bath. The reaction mixture was extracted with 4 mL of toluene, and the test tubes were vortexed for 20 s and cooled. Thereafter, the light absorbance at 520 nm was measured by using a UV–VIS spectrophotometer (Hitachi U-2910, Tokyo, Japan). The free proline content was determined on the basis of the standard curve at 520 nm absorbance.

### Determination of oxidative stress markers

The degree of lipid peroxidation was evaluated as the malondialdehyde (MDA) content. Briefly, 0.1 g of frozen leaves were ground at 4°C in a mortar with 25 mL of 50  mM phosphate buffer solution (pH 7.8) containing 1% polyethene pyrrole. The homogenate was centrifuged at 10,000 × *g* at 4°C for 15 min. The mixtures were heated at 100°C for 15–30 min and then quickly cooled in an ice bath. The absorbance of the supernatant was recorded by using a spectrophotometer (xMark™ microplate absorbance spectrophotometer; Bio-Rad, United States) at wavelengths of 532, 600, and 450 nm. Lipid peroxidation was expressed as L mol g^−1^ using the following formula: 6.45 (A532 A600) – 0.56 A450. Lipid peroxidation was measured using a method previously published by Heath and Packer.^51^

For the hydrogen peroxide (H_2_O_2_) assay, leaf and root samples were homogenized with 50 mM phosphate buffer at pH 6.5. After that, homogenized samples were centrifuged at 6000 × *g* for 25 min, followed by the addition of H_2_SO_4_ (20% v/v) and again centrifuged at 6000 × *g* for 15 min H_2_O_2_ contents were estimated by taking the absorbance at 410 nm, and calculations were completed with the help of extinction coefficient (0.28 μmol^−1^ cm^−1^).^43^

### Determination of AsA-GSH cycle

Glutathione (GSH), glutathione disulfide (GSSH), DHA (dehydroascorbic acid), and ascorbic acid (AsA) were determined in fresh leaves,^52^ and were extracted by homogenizing 0.2 g of leaves in TCA and then collecting the supernatant by centrifugation. The GSH concentration was measured in a solution including phosphate buffer, supernatant, and DTNB reagent (PBS, pH 7.0). The amount of GSH was determined by a spectrophotometer. To measure the AsA content, NaH_2_PO_4_ solution, enzyme extract, distilled water, and 10% TCA were mixed to determine the concentration of AsA in the samples. After a 30-s incubation period, FeCl_3_ solution, H_3_PO_4_, and 2,2′-3-dipyridine were added to the reaction mixture. The FeCl_3_ and 2,2′-dipyridine reacted with AsA to produce a red-colored complex that can be measured spectrophotometrically at 525 nm. The amount of AsA present in the sample was calculated.

### Determination of Cd contents

Finely ground samples were digested with pure HNO3 at 190°C for 45 min (10 min pre-heating, 15 min heating, 20 min cooling) in a microwave oven (Mars 6, CEM Corporation, USA) with the settings described in details.^53^ The samples were diluted with 2% HNO_3_ and determined by inductively coupled plasma‒mass spectrometry (ICP‒MS; Agilent 7700, Agilent Technologies Inc., USA).

### Rhizosphere microbiome, health risk assessment, and molecular analysis

To assess microbial diversity in the rhizosphere, soil samples were collected from the root zones of rice plants across the treatments. Total genomic DNA was extracted using a commercial DNA isolation kit. The bacterial community structure was examined by amplifying the 16S rRNA gene's V3–V4 hypervariable regions using region-specific primers, followed by sequencing on the Illumina MiSeq platform. Sequencing depth was normalized across all samples by rarefying to the minimum read count to ensure accurate and unbiased microbial diversity comparisons. This approach enables the evaluation of microbial richness, diversity, and community composition, as described by Henckel et al.^54^

To estimate potential human exposure to toxic elements, HRI and DIM were determined using formulas that incorporate measured metal concentrations in plant tissues, average daily food consumption, and standardized body weight assumptions. The concentrations of cadmium and other metals were quantified in acid-digested, oven-dried shoot samples using atomic absorption spectrophotometry (AAS), based on the protocol outlined by Sanaei et al.^55^

For proteomic analysis, total proteins were extracted from liquid nitrogen-frozen leaf tissues using a phenol-based extraction method. Protein concentrations were determined using the Bradford assay. SDS‒PAGE was used to separate the isolated proteins, which were analyzed through mass spectrometry to identify key stress-responsive proteins following the approach of Bhushan et al.^56^

To examine gene expression patterns, total RNA was isolated from leaves using TRIzol reagent. The quality and purity of RNA were verified using a NanoDrop spectrophotometer. Quantitative real-time PCR (qRT‒PCR) was performed using gene-specific primers to quantify the expression levels of DEGs and stress-related genes. These molecular insights were used to correlate.

### Statistical analysis

All the data in this study were given as arithmetic means analyzed by Statistix 8.1. The values are the means of four replications, and one-way analysis of variance (ANOVA) was used (*P* ≤ 0.05) to evaluate the influence of photo technology on contaminated soil remediation. The mean between different treatments was analyzed using Fisher's highest significant difference (HSD) test. Graphical representation was conducted using Origin-2017, while Pearson correlation and heat map analysis were carried out in R Studio to assess the relationships among physiological and biochemical traits.

## Results

### Effect of salinity and cadmium stress and their alleviation using PGPR and salicylic acid (SA) on morphological, photosynthetic, and gas exchange attributes

In the present study, different growth parameters, photosynthetic pigments, and also the gas exchange parameters in *O. sativa* under salinity and Cd stress with the application of SA and PGPR were measured. The growth and biomass data are presented in Table 1, and the photosynthetic pigments and gas exchange characteristics are presented in Table 2. According to the given results, salinity and Cd stress caused a significant (*P* < 0.05) toxicity in *O. sativa* and decreases plant height, shoot length, shoot fresh weight, root length, root fresh weight, shoot dry weight, root dry weight, chlorophyll-a, chlorophyll-b, total chlorophyll, carotenoid content, stomatal conductance, net photosynthesis, transpiration rate, and intercellular CO₂ concentration compared to control. However, the application of SA and PGPR caused increase in plant height, shoot length, shoot fresh weight, root length, root fresh weight, shoot dry weight, root dry weight, chlorophyll-a, chlorophyll-b, total chlorophyll, carotenoid content, stomatal conductance, net photosynthesis, transpiration rate, and intercellular CO₂ concentration compared to the plants which were grown in the control. The application of SA and PGPR, when applied to the plants which were not treated with salinity and Cd stress, there was a significant (*P *< 0.05) increase in plant height, shoot length, shoot fresh weight, root length, root fresh weight, shoot dry weight, root dry weight, chlorophyll-a, chlorophyll-b, total chlorophyll, carotenoid content, stomatal conductance, net photosynthesis, transpiration rate, and intercellular CO₂ concentration was observed in the plants compared to those which were not treated with the application of SA and PGPR. Although the combined application of SA and PGPR induced higher growth and biomass, compared to the individual applications of SA and PGPR.

**Figure 2.**
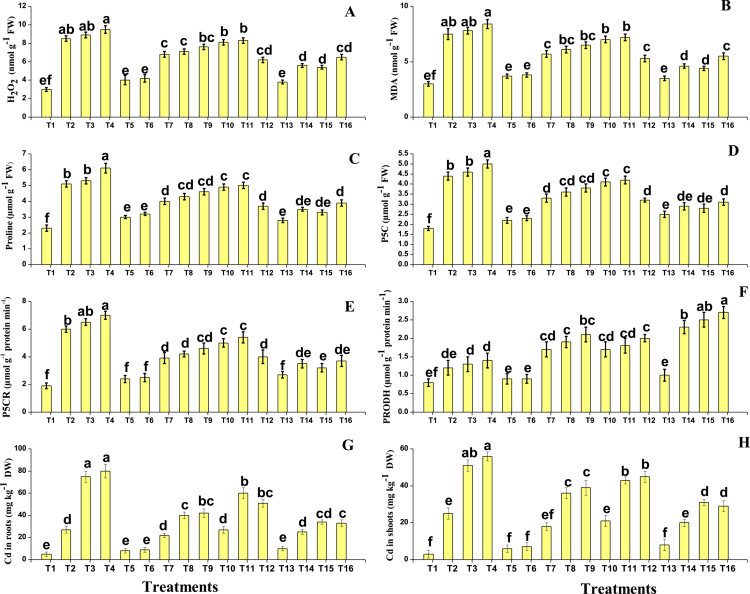
Effect of different treatments on (A) anthocyanin, (B) total sugar, (C) total flavonoids, (D) total phenolics, (E) ascorbic acid (ASA), (F) glutathione (GSH), (G) oxidized glutathione (GSSG), and (H) dehydroascorbate (DHA) content in *Oryza sativa* under salinity and Cd stress. Error bars represent the standard error (SE) of three replicates. Different letters above the bars indicate significant differences at *P* < 0.05 (Tukey's HSD test).

**Table 1.** Effect of salinity and cadmium stress and their mitigation using PGPR and salicylic acid (SA) on morphological traits of rice (*Oryza sativa* L.) at 45 d after sowing (DAS).

Treatments	Treatment description	Plant height (cm)	Shoot length (cm)	Root length (cm)	Shoot fresh weight (g)	Root fresh weight (g)	Shoot dry weight (g)	Root dry weight (g)
T1	Control (no stress)	62.0 ± 1.7^bc^	52.0 ± 1.4^b^	11.1 ± 0.4^b^	12.1 ± 0.4^b^	3.2 ± 0.12^b^	3.8 ± 0.19^b^	1.1 ± 0.13^b^
T2	Salinity stress	43.0 ± 1.1^e^	36.0 ± 1.0^e^	8.5 ± 0.3^e^	7.5 ± 0.2^e^	2.3 ± 0.14^e^	2.0 ± 0.16^e^	0.6 ± 0.15^e^
T3	Heavy metal stress	42.8 ± 1.1^e^	34.9 ± 1.0^ef^	8.3 ± 0.3^e^	7.2 ± 0.2^e^	2.2 ± 0.15^e^	1.9 ± 0.15^e^	0.6 ± 0.13^e^
T4	Salinity + Cd	40.2 ± 1.0^ef^	32.6 ± 0.9^f^	7.6 ± 0.2^f^	6.5 ± 0.2^f^	2.0 ± 0.16^f^	1.7 ± 0.14^f^	0.5 ± 0.16^e^^f^
T5	PGPR (alone)	67.2 ± 1.9^ab^	55.0 ± 1.7^b^	12.2 ± 0.4^b^	13.6 ± 0.5^ab^	3.8 ± 0.18^ab^	4.2 ± 0.11^ab^	1.4 ± 0.18^ab^
T6	Phytohormone (SA alone)	66.1 ± 1.8^ab^	54.0 ± 1.5^ab^	11.8 ± 0.4^b^	13.0 ± 0.4^b^	3.6 ± 0.18^b^	4.0 ± 0.12^b^	1.3 ± 0.13^b^
T7	PGPR + salinity	54.0 ± 1.4^c^	44.0 ± 1.2^c^	9.8 ± 0.3^c^	9.7 ± 0.3^c^	2.9 ± 0.15^c^	2.8 ± 0.13^c^	0.9 ± 0.18^c^
T8	PGPR + Cd	52.6 ± 1.3^c^	42.6 ± 1.2^c^	9.5 ± 0.3^c^	9.4 ± 0.6^cd^	2.8 ± 0.14^c^	2.7 ± 0.17^c^	0.8 ± 0.15^c^
T9	PGPR + salinity + Cd	48.2 ± 1.2^d^	38.5 ± 1.1^d^	8.9 ± 0.3^d^	8.3 ± 0.3^d^	2.5 ± 0.13^d^	2.3 ± 0.16^d^	0.7 ± 0.14^d^
T10	SA + salinity	51.0 ± 1.3^cd^	41.0 ± 1.2^cd^	9.3 ± 0.3^cd^	9.1 ± 0.5^cd^	2.7 ± 0.12^cd^	2.6 ± 0.15^cd^	0.8 ± 0.13^cd^
T11	SA + Cd	49.5 ± 1.2^d^	39.6 ± 1.1^d^	9.0 ± 0.3^d^	8.7 ± 0.3^d^	2.6 ± 0.12^d^	2.4 ± 0.18^d^	0.8 ± 0.12^d^
T12	SA + salinity + Cd	46.5 ± 1.1^de^	37.4 ± 1.1^de^	8.7 ± 0.3^de^	7.9 ± 0.2^de^	2.4 ± 0.16^de^	2.2 ± 0.14^de^	0.7 ± 0.17^de^
T13	PGPR + SA	68.5 ± 1.6^a^	56.2 ± 1.3^a^	13.0 ± 0.4^a^	13.9 ± 0.4^a^	3.9 ± 0.15^a^	4.3 ± 0.12^a^	1.5 ± 0.18^a^
T14	PGPR + SA + salinity	58.2 ± 1.5^bc^	47.4 ± 1.3^c^	10.4 ± 0.4^c^	10.6 ± 0.4^c^	3.1 ± 0.18^c^	3.2 ± 0.15^c^	1.0 ± 0.11^c^
T15	PGPR + SA + Cd	56.5 ± 1.4^c^	46.0 ± 1.2^c^	10.1 ± 0.4^c^	10.2 ± 0.4^c^	3.0 ± 0.11^c^	3.0 ± 0.12^c^	0.9 ± 0.15^c^
T16	PGPR + SA + salinity + Cd	60.0 ± 1.5^bc^	48.6 ± 1.3^bc^	10.6 ± 0.4^bc^	11.0 ± 0.4^bc^	3.2 ± 0.1^bc^	3.4 ± 0.11^bc^	1.0 ± 0.1^bc^

The values represent the mean ± SE (*n* = 3). Different letters within each column indicate significant differences among treatments according to Tukey's HSD test at *P* < 0.05.

**Table 2.** Effect of salinity and cadmium stress and their alleviation using PGPR and salicylic acid (SA) on chlorophyll content, carotenoids, and gas exchange traits of rice at 45 DAS.

Treatments	Treatment description	Total chlorophyll content (mg/g FW)	Carotenoid content (mg/g FW)	Pn (µmol m⁻² s⁻¹)	gs (mol m⁻² s⁻¹)	Tr (mmol m⁻² s⁻¹)	Ci (µmol mol⁻¹)
T1	Control (no stress)	2.40 ± 0.08^cd^	0.42 ± 0.02^c^	18.5 ± 0.5^b^	0.35 ± 0.03^b^	5.1 ± 0.2^b^	255 ± 5^ab^
T2	Salinity stress	1.60 ± 0.06^f^	0.28 ± 0.03^e^	11.2 ± 0.3^f^	0.20 ± 0.01^de^	3.2 ± 0.11^de^	248 ± 6^c^
T3	Heavy metal stress^7^	1.52 ± 0.05^f^	0.26 ± 0.01^e^	10.8 ± 0.3^f^	0.18 ± 0.04^e^	3.0 ± 0.1^e^	249 ± 5^c^
T4	Salinity + Cd	1.38 ± 0.04^fg^	0.23 ± 0.04^e^	9.2 ± 0.2^f^	0.15 ± 0.01^ef^	2.6 ± 0.12^f^	247 ± 4^c^
T5	PGPR (alone)	2.75 ± 0.09^b^	0.48 ± 0.03^b^	20.4 ± 0.5^ab^	0.40 ± 0.03^ab^	5.6 ± 0.2^ab^	260 ± 6^a^
T6	Phytohormone (SA alone)	2.65 ± 0.08^bc^	0.46 ± 0.05^bc^	19.8 ± 0.6^b^	0.38 ± 0.02^b^	5.4 ± 0.2^ab^	258 ± 3^a^
T7	PGPR + salinity	2.10 ± 0.07^de^	0.36 ± 0.04^cd^	15.5 ± 0.5^cd^	0.28 ± 0.01^c^	4.3 ± 0.14^c^	252 ± 7^ac^
T8	PGPR + Cd	2.05 ± 0.07^de^	0.35 ± 0.05^cd^	15.1 ± 0.3^cd^	0.27 ± 0.04^cd^	4.1 ± 0.17^c^	250 ± 4^bc^
T9	PGPR + salinity + Cd	1.88 ± 0.06 ^e^	0.32 ± 0.04^d^	13.5 ± 0.4^e^	0.24 ± 0.02^d^	3.6 ± 0.16^d^	248 ± 6^c^
T10	SA + salinity	2.00 ± 0.08^de^	0.34 ± 0.05^cd^	14.7 ± 0.5^d^	0.26 ± 0.06^d^	3.9 ± 0.16^cd^	253 ± 4^b^
T11	SA + Cd	1.95 ± 0.07^e^	0.33 ± 0.03^cd^	14.3 ± 0.6^de^	0.25 ± 0.01^d^	3.8 ± 0.13^cd^	252 ± 5^b^
T12	SA + salinity + Cd	1.75 ± 0.06^ef^	0.30 ± 0.01^d^	12.6 ± 0.2^de^	0.22 ± 0.02^de^	3.4 ± 0.17^d^	251 ± 5^bc^
T13	PGPR + SA	2.85 ± 0.08^a^	0.49 ± 0.02^a^	21.0 ± 0.4^a^	0.46 ± 0.03^a^	5.8 ± 0.2^a^	260 ± 7^a^
T14	PGPR + SA + salinity	2.25 ± 0.06^c^	0.40 ± 0.03^c^	16.2 ± 0.2^c^	0.30 ± 0.01^c^	4.5 ± 0.13^bc^	254 ± 6^ab^
T15	PGPR + SA + Cd	2.20 ± 0.07^d^	0.38 ± 0.01^cd^	15.9 ± 0.4^c^	0.29 ± 0.02^c^	4.3 ± 0.17^bc^	253 ± 8^b^
T16	PGPR + SA + salinity + Cd	2.35 ± 0.05^c^	0.42 ± 0.02^c^	17.0 ± 0.4^bc^	0.32 ± 0.01^bc^	4.8 ± 0.1^b^	256 ± 5^ab^

Values represent the mean ± SE (*n* = 3). Different letters within each column indicate significant differences among treatments according to Tukey's HSD test at *P* < 0.05.

### Effect of salinity and cadmium stress and their alleviation using PGPR and SA on oxidative stress, enzymatic antioxidants, and their respective gene expression

**Figure 3.**
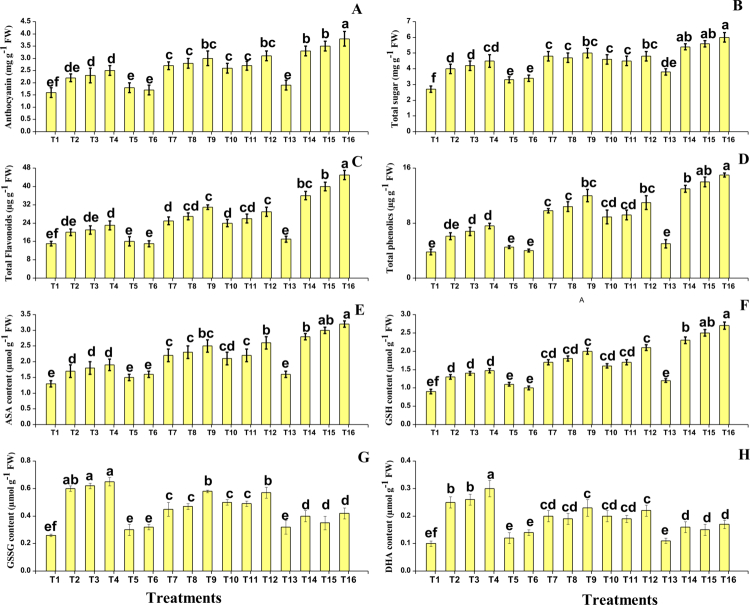
Effect of different treatments on (A) hydrogen peroxide (H₂O₂), (B) malondialdehyde (MDA), (C) proline, (D) pyrroline-5-carboxylate (P5C), (E) pyrroline-5-carboxylate synthase (P5CR), (F) proline dehydrogenase (PRODH), (G) cadmium (Cd) content in roots, and (H) Cd content in shoots of *Oryza sativa* under salinity and Cd stress. The values are expressed as the mean ± SE (*n* = 3). Different letters indicate significant differences (*P* < 0.05) based on Tukey's HSD test.

In the present study, different oxidative stress biomarkers, i.e., malondialdehyde (MDA) and hydrogen peroxide (H_2_O_2_), were measured from *O. sativa* as presented in Figure 3. According to the results, it was observed that the salinity and Cd stress caused a significant (*P* < 0.05) increase in MDA and H_2_O_2_ contents compared to the control. Although the application of SA and PGPR decreases the MDA and H_2_O_2_ contents in *O. sativa*. We have also noticed that the individual application of SA and PGPR also decreased the MDA and H_2_O_2_ contents when the plants were grown without the contamination of salinity and Cd in the soil. In addition, the combined application of SA and PGPR showed more severe results when compared to the plants which grown in the alone application of SA and PGPR. Different enzymatic antioxidants, i.e., superoxidase dismutase (SOD), ascorbate peroxidase (APX), peroxidase (POD), catalase (CAT), and also their relevant gene expression, i.e., SOD, POD, CAT, and APX, were measured in the tissues of *O. sativa*. The results regarding the enzymatic antioxidants and their relevant gene expression are presented in Table 3. According to the results, we have noticed that the salinity and Cd stress in the soil significantly (*P* < 0.05) increased the enzymatic antioxidants, i.e., SOD, POD, CAT, and APX, and their relevant gene expression compared to the plants grown in the control treatment. The present findings also showed that the alone application of either SA or PGPR also increased the activities of SOD, POD, CAT, and APX, and their relevant gene expression compared to the plants that were not treatment with the application of SA and PGPR salinity and Cd stressed soil. In addition, the maximum activities of SOD, POD, CAT, and APX and their relevant gene expression were observed in the plants that were grown in the combined application of SA and PGPR.

**Table 3.** Antioxidant enzyme activities and related gene expression in rice under different treatments at 45 DAS.

Treatments	Treatment description	SOD (U mg⁻¹ protein)	POD (U mg⁻¹ protein)	CAT (U mg⁻¹ protein)	APX (U mg⁻¹ protein)	SOD gene (fold)	POD gene (fold)	CAT gene (fold)	APX gene (fold)
T1	Control	21.5 ± 0.4^g^	18.0 ± 0.5^fg^	9.0 ± 0.4^f^	5.2 ± 0.2^ef^	1.2 ± 0.12^ef^	1.1 ± 0.13^f^	1.0 ± 0.15^f^	0.9 ± 0.14^f^
T2	Salinity	26.2 ± 0.3^e^	23.5 ± 0.6^e^	12.4 ± 0.38^d^	7.4 ± 0.3^d^	1.7 ± 0.19^d^	1.5 ± 0.15^de^	1.3 ± 0.16^de^	1.1 ± 0.13^de^
T3	Cadmium	26.8 ± 0.8^e^	24.0 ± 0.6^e^	12.8 ± 0.6^d^	7.6 ± 0.3^d^	1.8 ± 0.17^d^	1.6 ± 0.12^d^	1.4 ± 0.14^d^	1.2 ± 0.16^d^
T4	Salinity + Cd	27.3 ± 0.5^de^	24.6 ± 0.6^e^	13.2 ± 0.56^d^	8.0 ± 0.3^d^	1.9 ± 0.12^d^	1.7 ± 0.11^d^	1.5 ± 0.13^d^	1.3 ± 0.14^d^
T5	PGPR alone	25.4 ± 0.9^f^	22.1 ± 0.5^f^	11.8 ± 0.49^e^	6.9 ± 0.3^e^	1.6 ± 0.15^e^	1.4 ± 0.11^ef^	1.2 ± 0.12^ef^	1.0 ± 0.14^ef^
T6	SA alone	24.9 ± 1.1^f^	21.4 ± 0.5^f^	11.4 ± 0.59^e^	6.5 ± 0.3^e^	1.5 ± 0.12^e^	1.3 ± 0.18^ef^	1.1 ± 0.16^ef^	1.0 ± 0.17^ef^
T7	PGPR + salinity	30.5 ± 0.6^d^	27.8 ± 0.6^d^	15.3 ± 0.63^cd^	9.6 ± 0.3^cd^	2.4 ± 0.17^cd^	2.2 ± 0.13^cd^	1.9 ± 0.14^cd^	1.6 ± 0.15^cd^
T8	PGPR + Cd	31.2 ± 0.6^cd^	28.5 ± 0.6^cd^	15.9 ± 0.4^cd^	10.1 ± 0.3^b^	2.6 ± 0.18^c^	2.4 ± 0.14^c^	2.0 ± 0.15^c^	1.7 ± 0.16^c^
T9	PGPR + salinity + Cd	32.6 ± 0.6^c^	29.7 ± 1^c^	16.9 ± 0.6^bc^	10.9 ± 0.3^b^	2.9 ± 0.13^b^	2.7 ± 0.12^b^	2.3 ± 0.13^b^	2.0 ± 0.16^b^
T10	SA + salinity	28.9 ± 0.6^d^	26.3 ± 0.7^d^	14.3 ± 0.5^cd^	8.9 ± 0.3^cd^	2.1 ± 0.15^cd^	1.9 ± 0.19^cd^	1.7 ± 0.16^cd^	1.4 ± 0.17^cd^
T11	SA + Cd	29.6 ± 0.6^d^	27.0 ± 0.8^cd^	14.7 ± 0.48^cd^	9.2 ± 0.3^c^	2.2 ± 0.17^cd^	2.0 ± 0.17^cd^	1.8 ± 0.18^cd^	1.5 ± 0.15^cd^
T12	SA + salinity + Cd	32.0 ± 0.6^c^	29.0 ± 0.9^b^	16.5 ± 0.4^b^	10.6 ± 0.3^b^	2.8 ± 0.2^bc^	2.6 ± 0.11^bc^	2.2 ± 0.13^bc^	1.9 ± 0.14^bc^
T13	PGPR + SA	25.9 ± 0.5^d^	23.2 ± 0.5^d^	12.1 ± 0.42^d^	7.2 ± 0.3^d^	1.6 ± 0.13^d^	1.4 ± 0.12^d^	1.2 ± 0.14^d^	1.1 ± 0.15^d^
T14	PGPR + SA + salinity	33.5 ± 0.6^bc^	30.8 ± 0.4^b^	17.6 ± 0.39^b^	11.5 ± 0.3^b^	3.1 ± 0.12^b^	2.9 ± 0.15^b^	2.5 ± 0.14^b^	2.2 ± 0.16^ab^
T15	PGPR + SA + Cd	34.7 ± 0.6 ^ab^	32.0 ± 0.9^ab^	18.5 ± 0.34^ab^	12.3 ± 0.3^ab^	3.6 ± 0.1^b^	3.3 ± 0.13^ab^	2.8 ± 0.14^ab^	2.3 ± 0.15^a^
T16	PGPR + SA + salinity + Cd	36.0 ± 0.7^a^	35.0 ± 0.69^a^	20.0 ± 0.4^a^	14.0 ± 0.3^a^	4.2 ± 0.1^a^	3.6 ± 0.1^a^	3.0 ± 0.12^a^	2.3 ± 0.13^a^

The values represent the mean ± SE (*n* = 3). Different letters within each column indicate significant differences among treatments according to Tukey's HSD test at *P* < 0.05.

### Effect of salinity and cadmium stress and their alleviation using PGPR and SA on secondary metabolites and nonenzymatic antioxidants

In the present study, nonenzymatic compounds and secondary metabolites were also measured from the *O. sativa* under salinity and Cd stress. The AsA–GSH cycle, including the contents of glutathione, ascorbate, glutathione disulfide, and dehydroascorbic acid, is presented in Figure 2, and we have noticed that the salinity and Cd toxicity significantly (*P* < 0.05) decrease the content of glutathione, ascorbate, and dehydroascorbic acid while increasing the content of glutathione disulfide from the tissues of the *O. sativa*. However, the application of SA and PGPR increased the contents of glutathione, ascorbate, and dehydroascorbic acid compared to the plants that were grown without the application of SA and PGPR. Nonenzymatic compounds, i.e., total phenolics, flavonoids, anthocyanins, and total sugar, were also measured from the tissues of *O. sativa* and are presented in Figure 2. According to the results, we have noticed that the salinity and Cd stress in the soil significantly (*P* < 0.05) increased the nonenzymatic compounds, i.e., total phenolics, flavonoids, ascorbic acid, anthocyanins, and total sugar, compared to the plants grown in control treatment. The present findings also showed that the alone application of either SA or PGPR also increased the activity of total phenolics, flavonoids, ascorbic acid, anthocyanins, and total sugar, compared to the plants that were not treated with the application of SA and PGPR in salinity- and Cd-stressed soils. In addition, the maximum activity of nonenzymatic compounds, i.e., total phenolics, flavonoids, ascorbic acid, anthocyanins, and total sugar, were observed in the plants which grown with the combined application of SA and PGPR application.

### Effect of salinity and cadmium stress and their alleviation using PGPR and SA on proline metabolism, and cadmium accumulation

In the present study, proline-related attributes and the AsA GSH cycle were also measured from the *O. sativa* under the salinity and Cd stress. The proline-related attributes are presented in Figure 3. The proline-related parameters, such as proline, pyrroline-5-carboxylate, pyrroline-5-carboxylate reductase, and pyrroline-5-carboxylate dehydrogenase were also measured from the *O. sativa* tissue, and the results showed that the salinity and Cd toxicity induced a significant (*P* < 0.05) decrease in proline, pyrroline-5-carboxylate, and pyrroline-5-carboxylate reductase compared to the control except the pyrroline-5-carboxylate dehydrogenase. However, the application of SA and PGPR, either individually or in combined form induced a significant (*P* < 0.05) decrease in the content of proline, pyrroline-5-carboxylate, and pyrroline-5-carboxylate reductase compared to those plants that were grown without the application of SA and PGPR except the pyrroline-5-carboxylate dehydrogenase. The Cd concentration of the roots and shoots was also measured in the present study when *O. sativa* grown in Cd-stressed soil with individual or combined application of SA and PGPR. The data regarding the Cd concentration from the roots and shoots are presented in Figure 3. According to the results, we have noticed that the Cd concentration in the soil medium significantly (*P* < 0.05) increased the Cd concentration in the roots and shoots when compared to the control. The application of SA and PGPR further increased the concentration of Cd in the roots and shoots when compared to the plants which were grown in the Cd-treated plants without the application of SA and PGPR.

### Effect of salinity and cadmium stress and their alleviation using PGPR and SA on modulation of microbial, molecular, and proteomic responses

**Figure 4.**
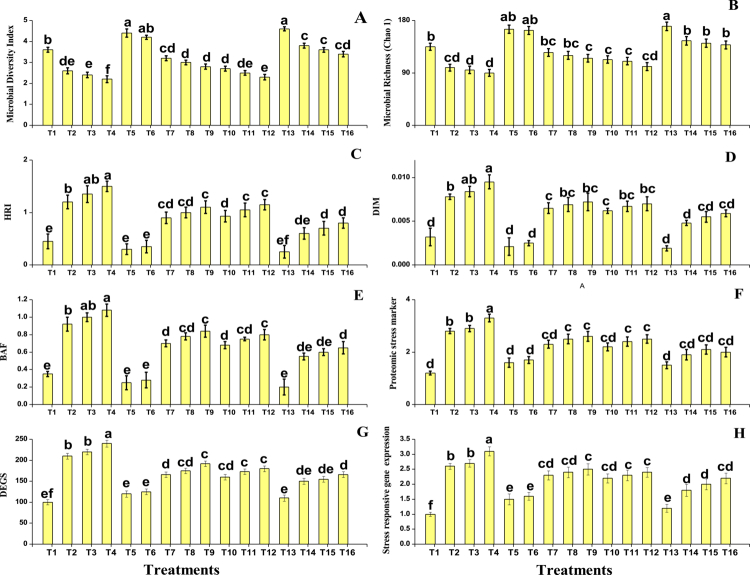
Effects of different treatments on (A) Microbial Diversity Index, (B) Microbial Richness, (C) Health Risk Index (HRI), (D) Daily Intake of Metals (DIM), (E) Bioaccumulation Factor (BAF), (F) Proteomic Stress Marker, (G) Differentially Expressed Genes (DEGs), and (H) Stress-responsive gene expression in *Oryza sativa* under combined salinity and cadmium stress. The values represent the mean ± SE (*n* = 3). Different letters denote statistically significant differences (*P* < 0.05) according to Tukey's HSD test.

The microbial, molecular, and proteomic responses were also measured in the present study from *O. sativa* grown under salinity and Cd stress, with the individual or combined application of PGPR and SA. The data regarding microbial richness, microbial index, and diversity indicators are presented in Figure 4. According to the results, salinity and Cd stress significantly (*P* < 0.05) decreased the microbial richness and microbial index when compared to the control. In contrast, diversity indicators such as host response index (HRI), diversity index of microbiota (DIM), and bioavailability factor (BAF) increased under stress conditions when compared to the control, indicating a shift in microbial community structure and an increase in the availability of toxic elements. The application of PGPR and SA further improved the microbial richness and microbial index, and also enhanced the microbial diversity metrics when compared to the plants grown under salinity and Cd stress without the application of PGPR and SA.

The data regarding stress-responsive gene expression, differentially expressed genes (DEGs), and proteomic stress markers are also presented in Figure 4. According to the results, the combined application of salinity and Cd stress markedly elevated the expression of stress-responsive genes, with a clear increase observed in transcriptional activity when compared to the control. The number of DEGs and the expression of proteomic stress markers also increased substantially under stress conditions. However, the application of PGPR and SA effectively moderated these molecular stress responses, as evident from a noticeable reduction in proteomic marker expression and DEG counts, along with a partial normalization of gene expression patterns when compared to the untreated stressed plants.

### Correlation analysis of physiological, biochemical, molecular, and microbial traits under combined salinity and cadmium stress

**Figure 5.**
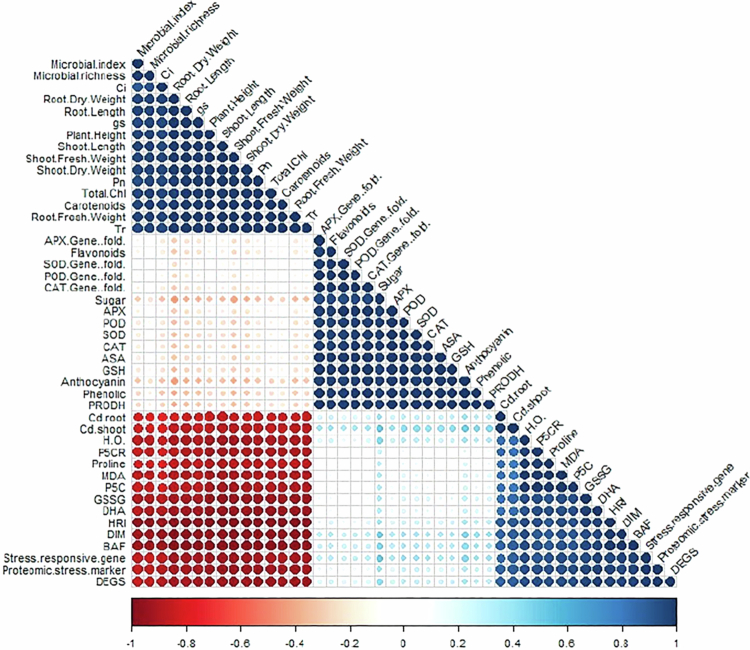
Pearson's correlation coefficients among the physiological, biochemical, molecular, and growth attributes of *Oryza sativa* under salinity and cadmium stress with treatments of PGPR (*Azospirillum brasilense*) and salicylic acid (SA). Positive correlations are represented in blue, and negative correlations are represented in red, with the size and intensity of the circles indicating the strength of the correlation.

A Pearson's correlation graph was constructed to quantify the relationship between various growth, physiological, biochemical, and microbial parameters with salinity and Cd accumulation in different parts of the plant (Figure 5). Salinity and Cd concentration in the roots were positively correlated with salinity and the Cd concentration in the shoots, SOD, peroxidase, proline, malondialdehyde, and microbial diversity indices such as DIM, HRI, and BAF, while negatively correlated with soluble sugar, shoot length, root dry weight, number of leaves, total chlorophyll content, transpiration rate, microbial richness, and the microbial index. Similarly, the salinity and Cd concentration in the shoots were positively correlated with the Cd concentration in the roots, SOD, peroxidase, proline, malondialdehyde, DIM, HRI, and BAF, while it was negatively correlated with soluble sugar, shoot length, root dry weight, number of leaves, total chlorophyll content, transpiration rate, microbial richness, and microbial index. This relationship highlights the strong interrelationship between plant physiological status, rhizosphere microbial health, and the accumulation of salinity and Cd in various plant tissues.

**Figure 6.**
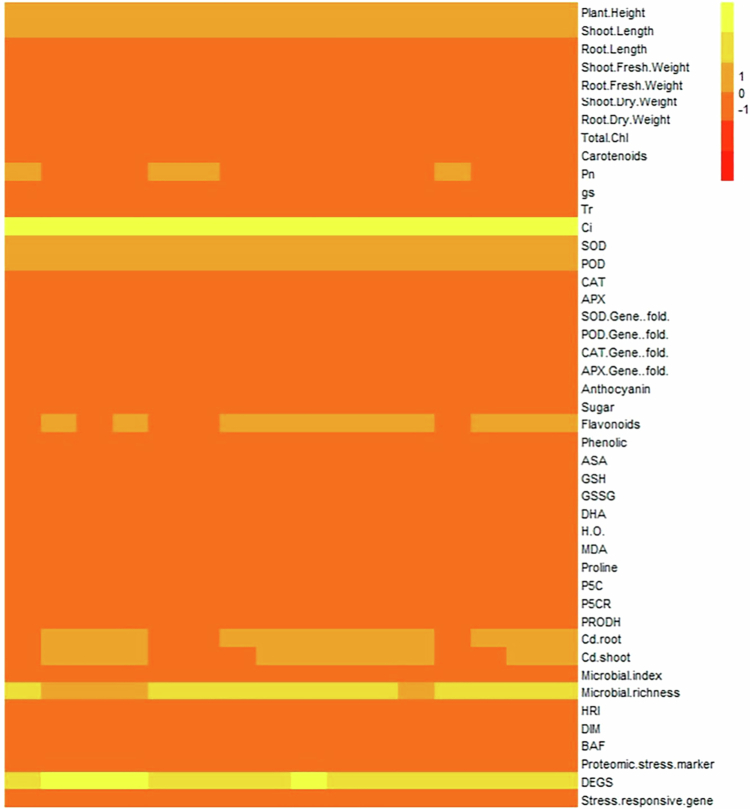
Heat map illustrating the normalized values of morphological, physiological, biochemical, molecular, and microbial traits in *Oryza sativa* across different stress and treatment combinations. Color intensity from red to yellow denotes the range from low to high expression or activity levels, highlighting treatment-induced modulation under combined salinity and cadmium stress conditions.

The heatmap analysis (Figure 6) provided additional visual confirmation of the strength and direction of relationships among the measured traits. Growth-related parameters such as shoot length, transpiration rate, and total chlorophyll showed strong positive co-variation with gas exchange attributes and antioxidant enzymes. In contrast, stress-related markers, including malondialdehyde, stress-responsive genes, proteomic stress markers, and Cd accumulation in plant tissues displayed negative associations with these growth indicators. Moreover, microbial richness and microbial index were closely associated with growth traits, whereas microbial diversity indices such as DIM, HRI, and BAF clustered alongside biochemical and molecular responses under stress conditions.

## Discussion

The individual application of SA and PGPR exhibited a significant effect on mitigating the negative effects of salinity and Cd stress in *O. sativa* plants, as shown by increasing plant growth and biomass, photosynthetic pigments, and gas exchange parameters. The use of SA and PGPR likely increased the plant's ability to maintain higher chlorophyll pigments by stabilizing chloroplast membranes and protecting the photosynthetic pigments from oxidative damage induced by salinity and Cd stress.^29^^,^^57^ The application of SA and PGPR, either individually or in combined form improved the net photosynthetic rate, stomatal conductance intercellular CO_2_ and transpiration rate, which may be attributed to their role in increasing carbon assimilation and maintaining the optimal stomatal apparatus, thereby ensuring effective gas exchange even under saline and metal stress conditions.^58^^,^^59^ SA may have played an important role in mitigating salinity and Cd stress by strengthening the plant's antioxidant system, modulating gene expression, and contributing to the reduced translocation and toxicity of Cd within plant tissues.^23^ This regulation likely contributed to the observed reduction in oxidative stress indicators such as MDA and H₂O₂ levels, as SA enhanced the antioxidant defense system and facilitated the scavenging of reactive oxygen species (ROS),^12^^,^^60^ thereby protecting cellular components from oxidative damage. Additionally, the increased activities of antioxidant enzymes, i.e., SOD, POD, CAT and APX, under SA application suggest that SA may increase the expression of genes related to stress attributes, thereby supporting the plant's antioxidant defense mechanisms against salinity and Cd-induced oxidative stress.^61^ *Azospirillum brasilense* is well known for its positive impact on increasing plant growth and biomass, particularly under stress conditions such as salinity and Cd stress, which is attributed to its ability to fix atmospheric nitrogen, produce phytohormones like indole−3-acetic acid (IAA), and modulate root structure, thereby improving nutrient and water uptake.^62^ The increasing levels of chlorophyll content observed with *A. brasilense* application can be primarily linked to its role in increasing nitrogen availability, a critical component of chlorophyll molecules, and its influence on the generation of cytokinins, which delay chlorophyll degradation and increase photosynthetic pigments under stress conditions.^63^ Moreover, *A. brasilense* plays a significant role in decreasing the generation of reactive oxygen species (ROS) by activating the activities of various enzymes, i.e., SOD, POD, CAT, and APX, and their relevant gene expression, and also the nonenzymatic compounds, i.e., total phenolics, flavonoids, ascorbic acid, anthocyanins, total sugar, and reducing sugar, which neutralize ROS and protect cellular components from oxidative damage.^64^

The positive impact of SA and PGPR on the AsA–GSH cycle further emphasizes their role in maintaining redox homeostasis within the plant cells.^33^^,^^65^ The increase in the content of GSH and AsA under individual application with SA and PGPR indicates that these treatments may have increased the regeneration capacity of the AsA–GSH cycle, thereby supporting high activities of enzymes to overcome ROS accumulation.^21^^,^^65^ This is further supported by the decrease in oxidized forms of these antioxidants, such as GSSG and DHA, which suggests that SA and PGPR helped the maintenance of a reduced cellular environment, which is crucial for overcoming oxidative damage.^9^ Cd uptake was significantly decreased in both roots and shoots under SA and PGPR applications, which can be attributed to the antioxidant-regulating action of SA and the metabolic modulation induced by SA, both of which likely contributed to reduced Cd translocation and accumulation in plant tissues.^66^ This reduction in Cd uptake is crucial for alleviating the negative impacts of Cd, thereby permitting the plant to maintain higher growth and other eco-physiological responses under stress conditions. The combined application of SA and PGPR showed even greater efficacy than their individual applications, indicating a potential synergistic effect.^67^ This combination further increased growth-related parameters, photosynthetic pigments, gas exchange parameters, antioxidant capacity, and, while significantly decreasing Cd uptake and oxidative stress biomarkers. The combined application between SA and PGPR likely improved the useful effects observed in their individual applications, suggesting that their combined use could be a strong approach for increasing plant tolerance to salinity and metal stress. These enhanced responses under combined treatment might also reflect hormetic effects, wherein low to moderate exposure to beneficial microbial and chemical stimuli activates plant defense and growth-promoting mechanisms beyond additive effects. Collectively, these findings suggest that the alleviation of salinity and Cd stress by PGPR and SA involves a multi-layered mechanism, wherein enhanced physiological traits (e.g., photosynthesis, stomatal conductance) are underpinned by improved redox balance through antioxidant enzyme activity, nonenzymatic metabolite accumulation, and upregulated expression of stress-responsive genes, ultimately ensuring cellular homeostasis and plant resilience. This highlights the potential of using SA and PGPR in combination to accomplish superior mitigation of salinity and metal toxicity in plants, paving the way for more effective bioremediation strategies in agriculture.

## Conclusion

The results of this study reveal the effects of salicylic acid (SA) and plant growth-promoting rhizobacteria (PGPR) application on salinity and cadmium-stressed *O. sativa*. We demonstrated that *O. sativa* is a metal-tolerant species, a characteristic conferred by its active antioxidant defense system. The application of SA and PGPR (individually or in combination) to salinity and cadmium-stressed plants improved plant growth and biomass, photosynthetic pigment levels, gas exchange attributes, sugars, ascorbate-glutathione cycle, proline metabolism, gene expression, proteomic responses, and rhizosphere microbial composition while alleviating oxidative stress and reducing oxidative stress in *O. sativa*. Notably, PGPR + SA significantly reduced Cd uptake and the health risk index. Furthermore, combined application of SA and PGPR resulted in more severe results and enhance plant tolerance compared to the single application. The treatment also upregulated antioxidant and stress-related gene expression and reshaped rhizosphere microbial diversity. Cd accumulation in roots and shoots was significantly reduced, and health risk index analysis confirmed decreased potential human toxicity. These findings suggest a novel integrative strategy to enhance rice resilience in contaminated soils. This is an initial investigation, and more research using different species in this field will be needed to identify the ideal dosages of various phytohormones in single and combination forms.

## Ethical approval

Not applicable.

## Consent to participate

Not applicable.

## Consent to publish

Written consent was sought from each author to publish the manuscript.

## Author contributions

Arwa Abdulkreem AL-Huqail conceptualized, acquired funding, supervised, and provided resources. Muna Abdul-Rahman Al-Malki and Dalia Mohammad Melebari were responsible for conducting the greenhouse experiment and collecting the samples. Hanan El Sayed Osman and Dikhnah Alshehri performed the biochemical and physiological analyses. Suliman Mohammed Suliman Alghanem conducted the gene expression analysis and interpreted the molecular data. Arwa Abdulkreem AL-Huqail and Amany H.A. Abeed wrote the original draft of the manuscript. All the authors contributed to data validation, reviewed and edited the manuscript critically for important intellectual content, and approved the final version for submission. Amany H.A. Abeed and Hesam Mousavi supervised the entire project and wrote the original draft of the manuscript.

## Data Availability

The data that support the findings of this study are available from the corresponding author upon reasonable request.
